# Amentoflavone ameliorates cold stress-induced inflammation in lung by suppression of C3/BCR/NF-κB pathways

**DOI:** 10.1186/s12865-019-0331-y

**Published:** 2019-12-30

**Authors:** Jiayi Cai, Chunyang Zhao, Yajie Du, Yuan Huang, Qingchun Zhao

**Affiliations:** 1 School of Life Sciences and Biopharmaceutis, Shenyang Pharmaceutical University, Shenyang, 110016 China; 2 Department of Pharmacy, General Hospital of Northern Theater Command, Shenyang, 110840 China; 3 College of Pharmaceutical Science, China Medical University, Shenyang, 110122 China; 4 Department of Pharmacy, the First Affiliated Hospital of China Medical University, Shenyang, 110001 China

**Keywords:** Amentoflavone, Cold stress, Inflammation, Complement C3, Lung

## Abstract

**Background:**

Cold stress, which may lead to local and systemic injury, is reported to be related to the immune system, especially the complement system. At present, the lack of effective treatment is a critical issue. Amentoflavone (AF), which can inhibit cold stress-induced inflammation in lung by multiple mechanisms, is the main therapeutic ingredient in plants of the genus *Selaginella*.

**Results:**

In the current study, we found that cold could induce lung inflammation related to the complement system and its downstream pathways. AF treatment significantly inhibited lung inflammation from cold exposure. We presented evidence that AF can bind to complement component 3 (C3) to regulate inflammation-related pathways involving Lck/Yes novel tyrosine kinase (Lyn), protein kinase B (Akt), nuclear factor-κB (NF-κB) and immune factors. Moreover, 30 mg/kg of AF caused significantly greater improvement than 15 mg/kg in reducing the level of C3 in lung tissue.

**Conclusions:**

AF can protect lung tissue from cold exposure. The protective effect may be achieved by inhibition of C3 and negative regulation of the B cell receptor (BCR)/NF-κB signaling pathways and high mobility group box 1 (HMGB1), which ultimately ameliorates the inflammatory response.

## Background

Cold stress, a severe and acute period of stress, may lead to local and systemic injury caused by the immune system [1]. As a common condition in high latitudes, cold stress produces damage in particular areas [2]. There are many hypotheses regarding how cold stress causes injury in target organs. Exposure to cold stress can directly cause hemodynamic changes, resulting in thrombosis, tissue ischemia and reperfusion, and inflammatory injury [3]. Therefore, cold-induced target organ damage occurs first in tissues with abundant blood flow. The lungs, as the main organs in the pulmonary circulation, provide oxygen for the whole body and are the most sensitive organs to changes in blood flow [4], especially changes in the state of the erythrocytes. In addition, the lungs are directly exposed to cold air, inducing smooth muscle contraction and even spasm, and are thus the first organs to be affected by cold exposure [5]. It has been proven that lung injury resulting from cold exposure is associated with poor prognosis and with prolonged recovery [6].

In previous studies, we have found that damage to lungs, may relate directly to a local inflammatory response induced by cold injury [4]. Thus, effective inhibition of the inflammatory response plays an important role in the treatment of frostbite patients and the improvement in patient prognosis. The pathogenesis of cold stress-induced inflammation is not fully understood at present, but it has been reported that the complement system plays a decisive role in the development of inflammation, and there is convincing evidence that inflammatory factors and immune-related pathways are involved [7, 8].

Currently, the treatment of cold stress includes first aid, systematic treatment and wound care [9]. Most of the available therapies are based on empirical treatment and lack sufficient support by evidence-based medicine. Although these treatments are effective with early detection, they require extensive recovery time and are associated with severe adverse events [10]. Thus, new therapeutic agents are needed to protect patients from injury caused by cold stress-induced inflammation.

Amentoflavone (AF), a polyphenol compound which widely exists in natural plants, is a major active ingredient of *Selaginella tamariscina* (Beauv.) Spring [11, 12]. This biflavonoid compound were reported to have anti-inflammation and anti-oxidation bioactivities in human and rats [13, 14]. It is known that hyper-activated complement system participates in acute lung injury (ALI) in rats [15]. Further study disclosed that AF enhanced the phosphorylation of PI3K (phosphatidylinositide 3-kinase), Akt (protein kinase B) and ERK1/2 (extracellular regulated protein kinases) down-regulated by MPP^+^ (1-methyl-4-phenylpyridinium) in human neuroblastoma (SH-SY5Y) cells [16]. AF acted as an inhibitor of nitric oxide synthase and reduces IκB (the inhibitor of NF-κB) phosphorylation, thus inhibiting NF-κB pathway [17, 18]. AF could adjust the level and activity of immune-related factors; for example, AF inhibited cyclooxygenase-2 (COX-2) and led to a decline in prostaglandin E2 (PGE2) content [19]. Molecular docking technology has revealed that AF and C3 have complementary structures that enable interaction. Computer simulations also provide fundamental evidence for further verification of the effects of AF.

Although the activity of AF has attracted a considerable amount of interest, the mechanism and molecular target remain unclear [20]. Given the therapeutic potential of this compound, we were eager to investigate whether AF could ameliorate cold stress-induced inflammation and its potential mechanisms in lung tissue in a murine model.

## Results

### Observations of general state

Compared with control group, rats in model group showed lower weight gain, higher lung/body weight ratios, duller hair, greater cyanosis of the tail, and significantly reduced food intake. Compared with model group, rats in the AF group had a lower occurrence of tail cyanosis, demonstrated an improvement in body weight and food intake, and exhibited reduced lung/body weight ratios. (Additional file 1).

### Local blood flow perfusion

**Fig. 1:**
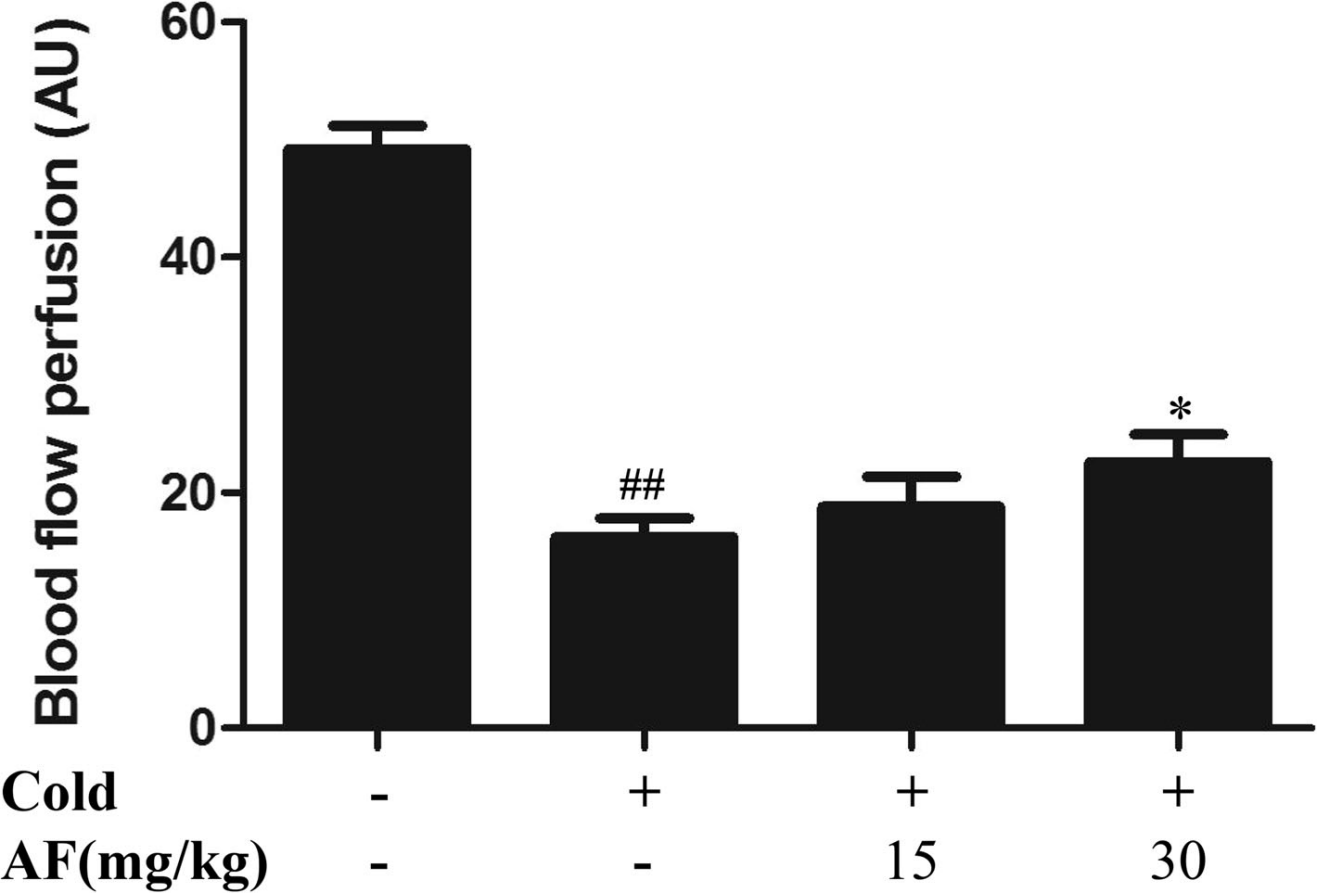
AF elevated the level of local blood flow perfusion in cold stress-treated rats (*n* = 6 per group). The data are presented as the means ±SD. * *P* < 0.05 vs. cold group, ## *P* < 0.01 vs. control group

Our study evaluated the effects of different doses of AF on local blood flow perfusion by using laser Doppler flowmetry in rats with cold stress. The blood perfusion values of the hind paws were calculated in Fig. 1. Compared with that in the control group, blood flow perfusion in the cold exposure group was significantly reduced (49.20 ± 4.93, *P* = 0.000). Compared with the model group, the group receiving the 30 mg/kg AF prevention experienced a significant restoration of local blood flow (22.65 ± 5.64, *P* = 0.046).

### Hemorheological indexes

In this study, several hemorheological indexes, including whole blood viscosity at a shear rate of 200/s, whole blood viscosity at a shear rate of 30/s, whole blood viscosity at a shear rate of 1/s, erythrocyte aggregation index, erythrocyte rigidity index and yield stress, were monitored for observation of hemodynamic change (Table 1). Compared with the control group, the cold stress group showed significant increases in blood viscosity (*P =* 0.020, 0.020 and 0.024), erythrocyte aggregation index (*P =* 0.017), erythrocyte rigidity index (*P =* 0.045) and yield stress (*P =* 0.028). Furthermore, compared with the model group, the group that received 15 mg/kg AF showed significant decreases in the erythrocyte aggregation index (*P* = 0.041), while the group that received 30 mg/kg AF showed significant decreases in whole blood viscosity (*P* = 0.041), erythrocyte rigidity index (*P* = 0.043) and yield stress (*P* = 0.001).

**Table 1:** AF decreased the levels of hemorheological indexes in cold stress-treated rats (n = 6 per group)

Group	Whole Blood Viscosity, 200/s mPa·S	Whole Blood Viscosity, 30/s mPa·S	Whole Blood Viscosity, 1/s mPa·S	Erythrocyte Aggregation Index	Erythrocyte Rigidity Index	Yield Stress mPa
Control	2.99 ± 0.44	3.98 ± 0.52	15.47 ± 1.45	5.21 ± 0.44	4.23 ± 1.16	5.63 ± 0.58
Model	4.85 ± 1.73^#^	7.13 ± 3.01^#^	37.36 ± 22.19^#^	7.22 ± 1.83^#^	8.26 ± 4.54^#^	17.90 ± 12.92^#^
AF (15 mg/kg)	3.55 ± 0.64	4.77 ± 0.86	19.28 ± 4.30	5.46 ± 0.79*	4.72 ± 1.94	7.31 ± 2.23
AF (30 mg/kg)	3.40 ± 0.80	4.61 ± 1.00	19.13 ± 3.66*	5.72 ± 0.82	3.83 ± 1.40*	7.44 ± 1.63*

* *P* < 0.05 vs. cold group, ^#^ *P* < 0.05 vs. control group. The data are presented as the means ±SD

### Effect of AF on lung histology

**Fig. 2:**
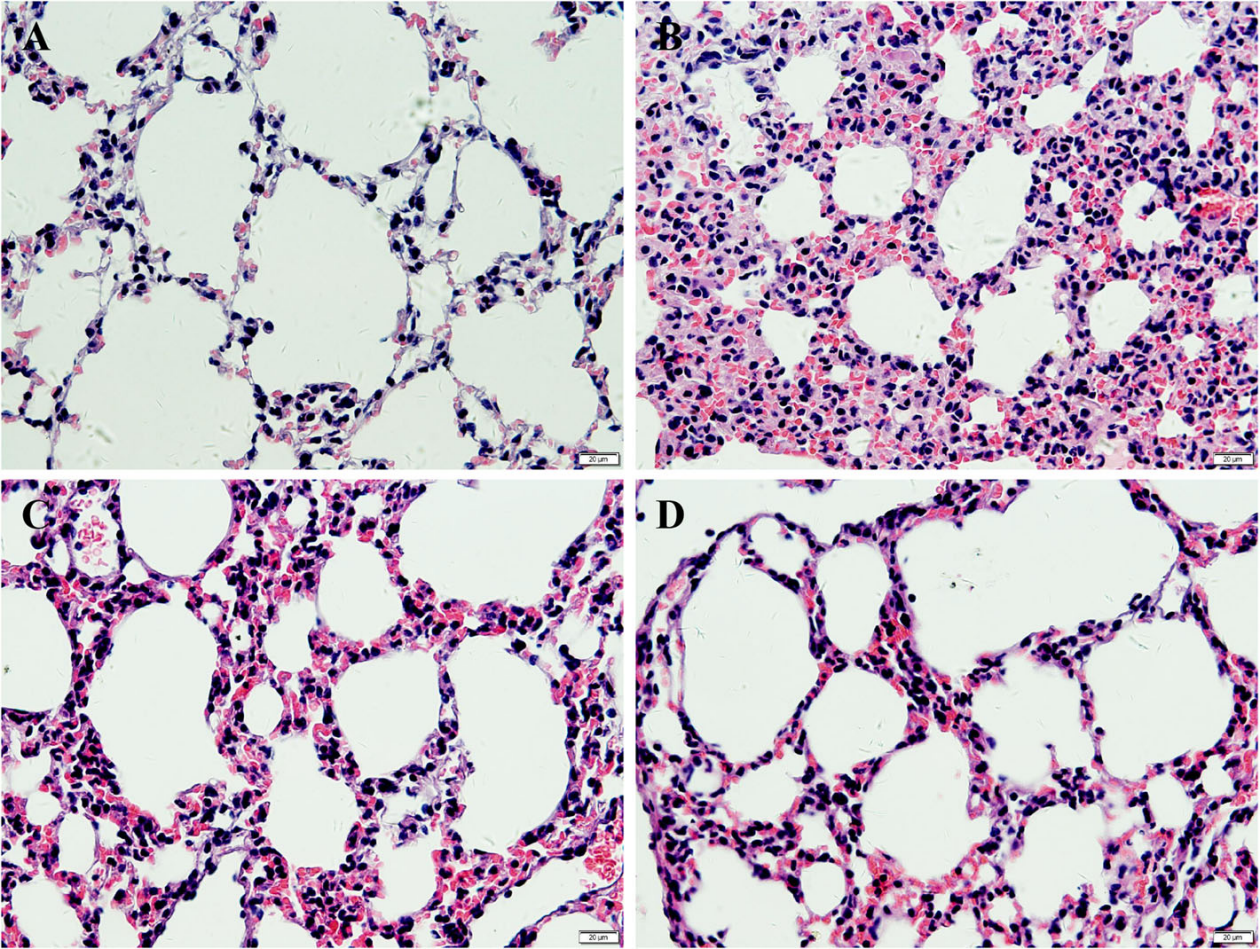
Effect of AF on pneumonocytes in lung tissue of cold stress-induced lung damage rates (n = 6 per group). Sections are 5 μm thick and photomicrographs are taken at 400×. **a** Normal control group; **b** Model control group; **c** and **d** Groups administrated with AF at a dose of 15 mg/kg and 30 mg/kg treatment group

In this study, the effect of AF on the cold stress-induced morphological changes in rat lung tissue was investigated by H&E (hematoxylin-eosin) staining (Fig. 2). Compared with the control group, the model group exhibited more edema and inflammatory cell infiltration in the alveolar cavity; the edema and infiltration were significantly relieved after AF treatment in dose-dependent manner. The results suggest that AF pretreatment has a protective effect against lung injury due to cold exposure.

### Effect of AF on the complement pathway

**Fig. 3:**
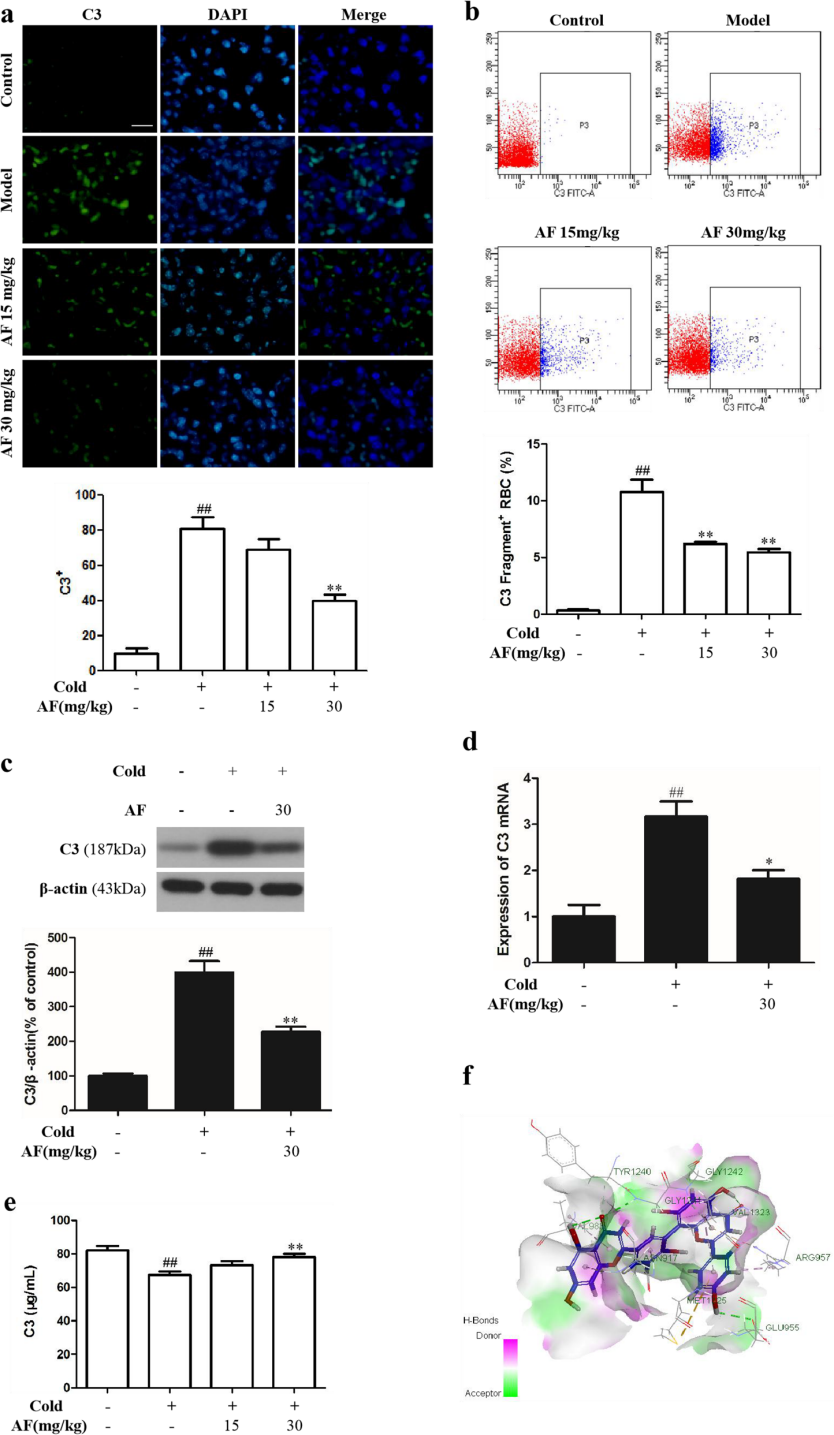
AF decreased the level of C3 in lung tissue of cold stress-treated rats (n = 6 per group). **a** Representative C3 staining in pneumonocytes by immunofluorescence and the ratio of C3-positive cells in each group. Sections are 5 μm thick and photomicrographs are taken at 400×. C3-positive cells were labeled by green fluorescence, DAPI restrained cell nuclei were labeled with blue fluorescence. **b** AF inhibited the level of C3 fragment deposition on hRBCs in cold stress-treated mice. Representative flow cytometry plots of C3 fragment deposition on hRBCs. Bar graphs showed mean values of three experiments. **c** AF reduced protein expression levels of C3 by western blotting. **d** AF reduced the gene expression of C3 in cold stress-treated rat by real-time PCR. **e** AF increased the level of C3 in serum of cold stress-treated rat. **f** The refined docking of C3-AF complex. The blue compound represents AF. The data are presented as the mean ± SD. ** *P* < 0.01, * *P* < 0.05 vs. cold group; ## *P* < 0.01 vs. control group

To investigate the effect of AF on complement activation after cold stress, immunofluorescence staining (Fig. 3a) and western blotting (Fig. 3c) were conducted. Compared with control group, the cold stress group rats demonstrated significant increases in C3 in lung tissue (*P* = 0.001 and 0.000). Pretreatment with 30 mg/kg AF significantly reduced the levels of C3 (*P* = 0.005 and 0.004). We used flow cytometry to detect the deposition of complement on the surface of erythrocytes in rats (Fig. 3b). There was not considerable C3 deposition on the surface of red blood cells (RBCs) in the control group. In contrast, C3 deposition increased significantly after cold stress (10.78 ± 2.16, *P* = 0.000), however, pretreatment with 15 mg/kg (6.18 ± 0.39, *P* = 0.006) and 30 mg/kg (5.45 ± 0.60, *P* = 0.003) AF significantly reduced the C3 levels on erythrocyte membranes, suggesting that AF might inhibit the activation of the complement pathway in lung tissue after cold exposure. Gene expression of C3 was measured using quantitative real time PCR (polymerase chain reaction, Fig. 3d). Significant higher levels of C3 were detected in cold stress group than the control (*P* = 0.003) and AF group (*P* = 0.012). Simultaneously, enzyme-linked immunosorbent assay (ELISA) was applied to assess the levels of C3 in rat serum (Fig. 3e). The model group showed significantly lower serum C3 than control group (67.37 ± 6.12, *P* = 0.000). Pretreatment with 30 mg/kg AF extract significantly reversed this effect (78.09 ± 5.48, *P* = 0.002). The refined docking of C3-AF complex was presented in Fig. 3f.

### Effect of AF on BCR/ NF-κB pathway

**Fig. 4:**
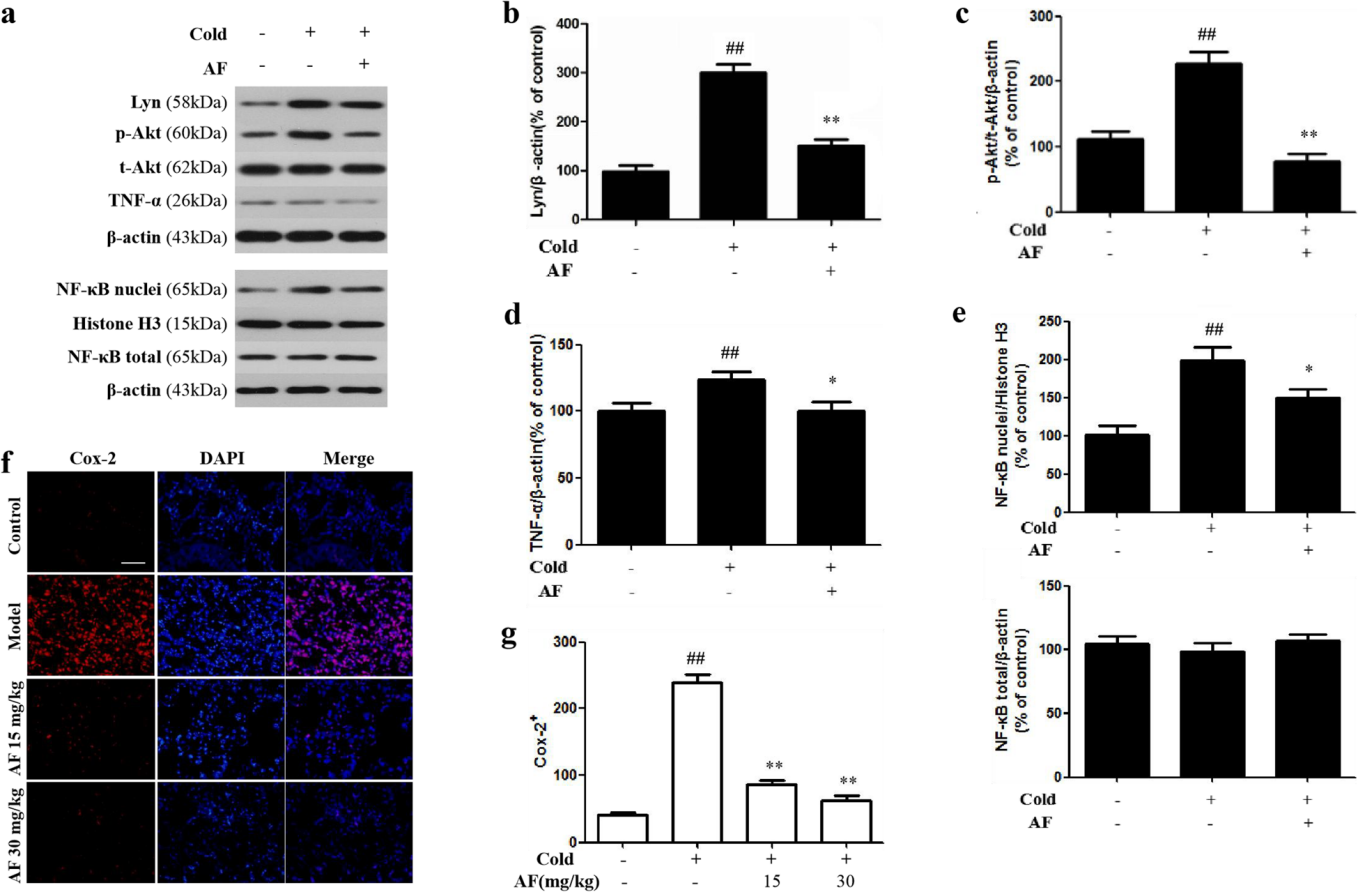
Effect of AF on BCR/NF-κB pathway in lung tissue of cold stress-induced rats (n = 6 per group). **a** Protein expression levels of Lyn, p-Akt, t-Akt, TNF-α, NF-κB p65 nuclei and NF-κB p65 total by western blotting. **b**-**e** Quantification of relative changes in protein expression. **f** AF decreased the level of COX-2 in lung tissue of cold stress-treated rats. Sections are 5 μm thick and photomicrographs are taken at 200×. Representative COX-2 staining in pneumonocytes by immunofluorescence. COX-2-positive cells were labeled by red fluorescence, DAPI restrained cell nuclei were labeled with blue fluorescence. **g** The ratio of COX-2-positive cells in each group. The data are presented as the mean ± SD. ** *P* < 0.01, * *P* < 0.05 vs. cold group; ## *P* < 0.01, # *P* < 0.05 vs. control group

To investigate the role of BCR/NF-κB pathway signaling in the observed effect of AF on cold stress induced lung injury, the western blotting method was applied to detect the expression of Lyn, t-Akt/p-Akt, NF-κB nuclei/NF-κB total and immune factors in the lung tissue of rats after cold stress and administration of AF (Fig. 4a). As shown in Fig. 4b-e, compared with the results in control group, the expression of Lyn (*P* = 0.013), p-Akt (*P* = 0.009), NF-κB nuclei (*P* = 0.037) and tumor necrosis factor-α (TNF-α, *P* = 0.040) in the lung tissues of the cold stress group rats was significantly elevated, and the expression of them in the rats that received 30 mg/kg AF was significantly reduced. Meanwhile, downregulation of t-Akt, NF-κB total were observed in the cold stress group. However, they were increased significantly after administration of AF. These observations indicated that inflammatory response occurred in lung tissue after cold stress and AF might inhibit BCR/NF-κB pathway to protect lung tissue.

In this study, the effect of AF on cold exposure-induced changes in COX-2 protein expression in lung tissue was investigated by immunofluorescence staining (Fig. 4f-g). The expression level of the COX-2 protein in the three experimental groups increased significantly compared with the levels in the control group (*P* = 0.000). Prevention with 15 mg/kg and 30 mg/kg AF significantly reduced the expression of COX-2 after frostbite (*P* = 0.000), suggesting that AF could inhibit the inflammatory reaction induced by cold exposure via downregulating COX-2.

### Effect of AF on HMGB1

**Fig. 5:**
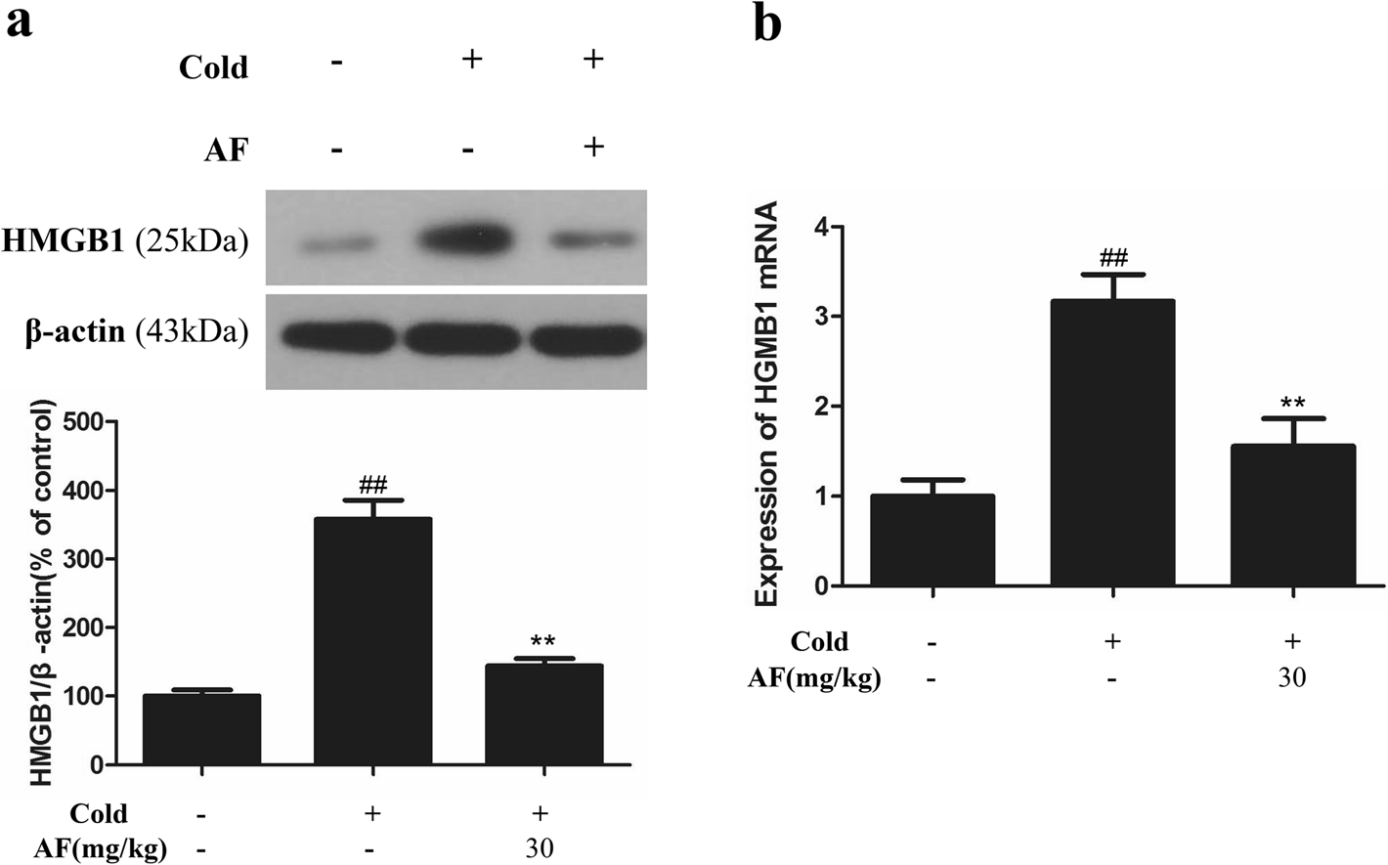
Effect of AF on HMGB1 in lung tissue of cold stress-induced rats (n = 6 per group). **a** AF reduced protein expression levels of C3 by western blotting. **b** AF reduced the gene expression of C3 in cold stress-treated rat by real-time PCR. ** *P* < 0.01 vs. cold group; ## *P* < 0.01 vs. control group

HMGB1 is an evolutionarily conserved non-histone chromatin-binding protein. During injury, activated immune cells and damaged cells release HMGB1 into the extracellular space, where HMGB1 functions as a proinflammatory mediator and contributes importantly to the pathogenesis of inflammatory diseases [21]. To investigate the effect of AF on HMGB1 in lung tissue after cold stress, western blotting (Fig. 5a) and real-time PCR (Fig. 5b) were performed. Compared with control group, the cold stress group rats demonstrated significant increases in HMGB1 (*P* = 0.000 and 0.002). Pretreatment with 30 mg/kg AF significantly reduced the effect (*P* = 0.001 and 0.010).

## Discussion

Cold environment may lead to local and systemic injury, however its potential mechanism remains unclear [1]. Previous studies suggested that, during cold stress, the crystallization of the extracellular fluid could directly damage the cell membrane [22] and as the temperature of the environment continues to decline, the intracellular liquid forms ice crystals and cells expand [23], affecting osmotic pressure, lead to changes in capillary permeability and exudation under rapid vasoconstriction [24]. Progress of thrombosis may cause damage to vessels and tissues, studies have confirmed the involvement of cytokines such as prostaglandins, bradykinin and histamine, which activate platelets and the coagulation cascade in the process of thrombosis [25]. Ischemia reperfusion injury after rewarming, results in inflammatory reactions, which share similarities with inflammation caused by burns [26, 27]. The degree of inflammation is easiest to observe in the feet, ear pinnae and other capillary-rich parts of the body [28]. To prevent heat loss, a series of compensatory responses occurs, including the weakening of the arterial pulse, the contraction of the body surface and the slowing of blood flow [29, 30]. In the current study, reduced local blood flow perfusion and increased hemorheological indexes were noticed in rats kept in a low temperature (− 20 °C) environment. These data are consistent with the proposal of the previous studies. Perelman et al. showed that, as a result of vasoconstriction, cold air inhalation could lead to sustained APW (amplitude of pulse wave) drop both during provocation and recovery period [31]. Keatinge et al. reported that, increases in platelet and blood viscosity were major factors in mortality from coronary and cerebral thrombosis in winter [32].

Complement activation leads to opsonization and phagocytosis by C3 fragment deposition, inflammation by recruitment of immune cells, endothelial and epithelial cells activation [33]. Accumulating evidence suggests the existence that, thrombin, human coagulation factors IXa, XIa, Xa, and plasmin were all found to effectively cleave C3 [34–37]. Complement Receptor 2 is expressed on B-cells interacting with C3d, binding iC3b or C3dg and forming a co-receptor complex with CD19/CD81, promoting B-cells activation [36]. In our study, higher C3 levels were noticed in cold injury rats, and the results are in agreement with previous studies. Mathews et al. presented evidence that, classical complement pathway activation in the cold was obtained by C4a, C3a, and C4d generation [38]. Takemura reported that, cold activation of complement resulted from circulating immune complexes composed mainly of immunoglobulin G (IgG) antibody [39].

We considered the key complement C3 as the target protein and downloaded the C3 protein structure from the RCSB PDB library. The molecular docking assessment with the C3 protein 2A73 was conducted to explore its potential interaction with AF [40]. Figure 3f shows the interaction between AF and the target protein. AF and C3 demonstrate suitable spatial matching and chemical compatibility. They form hydrogen bonds at GLU-955, GLY-1241, TYR-1240, GLY-1242 and ASN-917, and they experience van der Waals forces at VAL-983, MET-1235, ARG-957 and VAL-1323. These chemical bonds enable AF to bind effectively to C3 and might inhibit the occurrence of downstream immune responses. Therefore, the mechanism of AF is related to the complement system and is further related to anti-cold stress activity.

The BCR pathway plays a key role in B cell generation, activation and differentiation; this pathway, along with T-cell receptors (TCR), is involved in the recognition and activation of the precursor substances in the immune response [41]. Lyn, as a key tyrosine kinase in BCR pathway, was examined to verify the activation of BCR pathway in cold exposure injury. During the rewarming process, histiocyte apoptosis increases, and the Akt pathway is activated to inhibit cell apoptosis and promote cell growth, as NF-κB signal transduction [42]. In the current study, elevated Lyn, p-Akt and NF-κB nuclei levels were noticed in rats exposed to cold. These results are consistent with previous studies. Yang et al. confirmed that the BCR pathway is critical for the progress of cold stress-induced inflammation [43]. Research on cold panniculitis showed that the mechanism of cold stress was related to the BCR pathway and to B lymphocytes [44]. Ricklin et al. reported that downstream signaling induced inflammatory cells to cluster on the surface of target organs and vessels during the first 24 h after cold stress [45]. Banerjee found that NF-κB translocated to the nucleus and participates in the regulation of gene coding of inflammatory factors [18]. Some studies have found that in the heart, kidney, liver and other tissues, Akt phosphorylation levels increased significantly after cold stress, and these studies also detected the involvement of NF-κB, glycogen synthase kinase-3β (GSK-3β), cysteinyl aspartate specific proteinase-3 (Caspase-3), and the phosphorylation of Bad [46].

Human and rats have shared similar immune response process, therefore, rats are often used as research models when studying immune responses [4, 47]. Our experimental results revealed that AF could protect lung tissue from cold-exposed inflammation by inhibiting C3/BCR/NF-κB in rats, still, whether there is similar reaction in human body needs further research evidence. As multiple targets, including several proteins, are involved in the process of cold stress injury, further research is needed to fully describe the relationship between AF and cold stress-induced damage.

## Conclusions

In summary, AF can protect lung tissue from cold exposure. The protective effect may be achieved by the inhibition of complement C3 and the negative regulation of the BCR/NF-κB signaling pathways and HMGB1, which ultimately ameliorates the inflammatory response.

## Methods

### Animals

Male Sprague-Dawley rats (weight 200 ± 20 g) were purchased from Liaoning Changsheng Biotechnology Co., Ltd. [permit no. SCXK (Liao) 2015–0001; Benxi, China]. Experimental animal were maintained for 1 week in a controlled sterile environment under conditions of 24 ± 2 °C, 50% relative humidity and a 12 h light/dark cycle. Food pellets and tap water were available throughout the experiment. Animal care and handling procedures strictly followed the National Institutes of Health Guide for the Care and Use of Laboratory Animals (8th Edition) and were approved by the Institutional Animal Care and Use Committee of the General Hospital of Northern Theater Command (Shenyang, China).

### Drugs and model

Experimental animals were divided into three groups (*n* = 6 per group): (1) AF group, (2) control group, and (3) cold exposure model group. The administration group was treated with 15 or 30 mg/kg AF (double distilled water solution, *p.o.*; Chengdu Herbpurify, China) once per day for 2 weeks. The control and model groups were treated with equal volumes of double distilled water (*p.o*) once per day for 2 weeks. Four hours after the last administration, the model was established. Except for control group, the model and AF group rats were exposed to cold for one time, in a temperature-controlled box (− 20 °C for 8 h) [48]. Rats were placed in the box (two per box) before the cooling phase, and after the exposure period, rats were allowed to rewarm to room temperature before being removed from the box. The general state of the rats, including body weight, lung wet weight, food intake, hair and tail state, were observed and recorded during 2 weeks.

### Animal experiment and histological examination

At 24 h after the establishment of the model, rats were anesthetized with intraperitoneal injection of pentobarbital sodium (30 mg/kg). Blood samples were collected from the abdominal aorta, lungs were excised after cervical dislocation, and the rats were sacrificed. Fresh tissues for protein and gene analysis were frozen in liquid nitrogen. Samples for histological analysis were fixed in 10% formaldehyde and embedded in paraffin blocks. Sections were sliced using a microsystem microtome (Leica, Germany), and H&E staining was carried out.

### Blood flow perfusion and hemorheological indexes

Rats were anesthetized with pentobarbital sodium (30 mg/kg) at 20 h before the last administration, and the baseline perfusion values of the hind paws were measured by a laser Doppler flowmetry (Perimed, Sweden). Then, 4 hours after the last administration, rats were placed into a temperature-controlled box and held at − 20 °C for 8 h, processed as described previously. Four hours after the establishment of the model, rats were again anesthetized, and the local blood perfusion value of the hind paws was measured for comparison with baseline values. To measure the hemorheological indexes, blood samples were drawn from the abdominal aorta after Doppler flow detection, and the rats were sacrificed after intravenous injection of pentobarbital sodium (150 mg/kg). Blood samples were gently mixed, and the whole blood viscosity, erythrocyte aggregation index, erythrocyte deformability index and erythrocyte rigidity index were detected by a hemorheology analyzer (ZONCI, China).

### Western blotting analysis

Briefly, approximately 20 μg of protein was loaded into each gel well resolved by 10% Sodium dodecyl sulfate-polyacrylamide gel electrophoresis (SDS-PAGE). Polyvinylidene fluoride (PVDF) membranes were incubated overnight at 4 °C in tris buffered saline (TBS) containing 3% nonfat dry milk. Anti-Lyn, Anti-TNF-α, Anti-Akt, Anti-p-Akt and Anti-NF-κB p65 antibody were procured from WanleiBio, China. The blot was then washed and incubated with secondary antibody (WanleiBio, China). Antibody binding was detected by chemoluminescence staining using an electrochemiluminescence (ECL) detection kit (Beyotime Biotechnology, China). The density of each band was quantified by densitometry with Gel-Pro-Analyzer softweare.

### Immunofluorescence analysis

Immunostaining was conducted to identify the expression of C3 and COX-2 using a standard immunostaining kit (Abcam, USA). Antibodies against C3 and COX-2 were purchased from Abcam (USA) and used at a 1:100 dilution. Anti-C3 antibody used in our research recognizes both intact C3 and its cleaved products C3b, iC3b,C3d and C3dg. Immunohistochemical visualization was completed using 3,3-diaminobenzidine (DAB) (ZSGB-Bio, China) and counterstaining with hematoxylin solution. Negative controls with the omission of the primary antibodies were included and were simultaneously assessed.

### Elisa

The levels of complement C3 in serum were quantified using commercially available ELISA kits (Nanjing SenBeiJia Biological, China) according to the manufacturer’s instructions.

### RNA purification and real-time quantitative PCR analysis

Total RNA, isolated from native lung tissue using a Trizol kit (WLA088b, Wanlei, China), was tested by Ultraviolet Spectrophotometer (Thermo NANO 2000, USA). The purified target mRNA was reverse-transcribed to cDNA using first-strand cDNA synthesis Kit (WLA101a, Wanlei, China). Fluorescence quantitative analysis was conducted with 2 × PCR Master Mix (WLA046a, Wanlei, China) and SYBR Green I (SY1020, Solarbio, China) by Exicycler 96 analyzer (BIONEER, Korea). Real-time PCR was carried out as described previously [49]. Primers used in PCR for C3 and HMGB1 were as follows: C3 F (TCCCTGTATGTCTCCGTCAC), C3 R (AGCCTTTGCATTAGATCCCT), HMGB1 F (GGATATTGCTGCCTACAGAG) and HMGB1 R (CTTCATCCTCCTCATCATCT).

### Statistical analysis

All data values are expressed as the means ± SD. Statistical analysis was performed using one-way analysis of variance (ANOVA) followed by Dunnett’s test with GraphPad Prism 5.0 (GraphPad software, San Diego, CA). *P* values < 0.05 were considered statistically significant compared to the cold stress model or the control group.

## Supplementary information

**Additional file 1.** Assessment of inflammation due to cold stress.

## Data Availability

The datasets generated and/or analyzed during the current study are available from the corresponding author upon reasonable request.

## Abbreviations

AF: Amentoflavone
Akt: Protein kinase B
ALI: Acute lung injury
ANOVA: Analysis of variance
APW: Amplitude of pulse wave
BCR: B cell receptor
C3: Complement component 3
Caspase-3: Cysteinyl aspartate specific proteinase-3
COX-2: Cyclooxygenase-2
DAB: 3,3-diaminobenzidine
ECL: Electrochemiluminescence
ELISA: Enzyme-linked immunosorbent assay
ERK1/2: Extracellular regulated protein kinases
GSK-3β: Glycogen synthase kinase-3β
H&E stain: Hematoxylin-eosin stain
HMGB1: High mobility group box 1
IgG: Immunoglobulin G
IκB: The inhibitor of NF-κB
MPP^+^: 1-methyl-4-phenylpyridinium
NF-κB: Nuclear factor-κB
PAGE: Polyacrylamide gel electrophoresis
PCR: Polymerase chain reaction
PGE2: Prostaglandin E2
PI3K: Phosphatidylinositide 3-kinase
PVDF: Polyvinylidene fluoride
RBC: Red blood cell
SDS: Sodium dodecyl sulfate
SH-SY5Y: Human neuroblastoma
TBS: Tris buffered saline
TCR: T cell receptors
TNF: Tumor necrosis factor
