# Supplementary material for: Amentoflavone ameliorates cold stress-induced inflammation in lung by suppression of C3/BCR/NF-κB pathways
Source: BMC Immunol. 2019 Dec 30;20:49. doi: 10.1186/s12865-019-0331-y (PMC6937961; doi:10.1186/s12865-019-0331-y)
Supplement: Supplementary file 1 — Additional file 1. Assessment of inflammation due to cold stress. [file 12865_2019_331_MOESM1_ESM.docx]

**Additional file 1:**

**Assessment of inflammation due to cold stress**

|  | Control | Model | AF(15mg/kg) | AF(30mg/kg) |
| --- | --- | --- | --- | --- |
| Body weight (g) | 296.01±9.97 | 267.63±11.39^#^ | 282.63±10.38* | 291.13±5.82* |
| Lung wet weight (g) | 2.52±0.09 | 2.70±0.09^#^ | 2.69±0.10 | 2.48±0.05* |
| Lung/ body weight | 0.85% | 1.01%^#^ | 0.95% | 0.85%* |
| Food intake (g) | 24.25g±1.49 | 17.25g±1.04^#^ | 21.75±2.61* | 23.63±1.19* |
| Tail cyanosis Occurrence | 0/6 | 5/6 | 3/6 | 2/6 |
| Hair state | glossy | lusterless | lusterless | lusterless |

Table Observations of the general state of the rats

* *P*<0.05 vs. model group, ^#^ *P* < 0.05 vs. control group. The data are presented as the means±SD.
